# Supplementary material for: Prevalence of cancer related fatigue and its associated factors among adult cancer patients in eastern Ethiopia: A cross-sectional study
Source: PLoS One. 2026 Apr 24;21(4):e0347297. doi: 10.1371/journal.pone.0347297 (PMC13108774; doi:10.1371/journal.pone.0347297)
Supplement: S1 File — (DOCX) [file pone.0347297.s001.docx]

Questionnaire (English version) on the prevalence of CRF and its associated factors

Code no.__________________

| **PART I: SOCIO-DEMOGRAPHIC CHARACTERISTICS** | | | |
| --- | --- | --- | --- |
| 101 | Address | | 1. Rural  2. Urban |
| 102 | Age | | ___________ in years |
| 103 | Sex | | 1. Male  2. Female |
| 104 | Religion | | 1. Muslim  2. Orthodox  3. Protestant  4. Other (please, specify):________________ |
| 105 | Marital status | | 1. Single  2. Married  3. Divorced/Separated  4. Widowed/Widower |
| 106 | Educational level | | 1. No formal education  2. Primary school  3. Secondary school  4. High school and above |
| 107 | Occupational status | | 1. Private  2. Employed  3. Farmer  4. Merchant  5. Housewife  6. Other (please, specify)___________ |
| 108 | Household monthly income per capita | | _____________ in ETB |
| 109 | Medical payments | | 1. Self pay/out of pocket  2. Free /Public expense/employer |
| 110 | Social support: *Oslo social support scale (OSSS-3)* | |  |
| 110.1 | How many people are so close to you that you can count on them if you have great personal problems? | | 1. None  2. One-two  3. Three-five  4. More than five |
| 110.2 | How much interest and concern do people show in what you do? | | 1. None  2. Little  3. Uncertain  4. Some  5. A lot |
| 110.3 | How easy is it to get practical help from neighbors if you should need it? | | 1. Very difficult  2. Difficult  3. Possible  4. Easy  5. very easy |
| **PART II: PHYSICAL AND BEHAVIORAL FACTORS** | | | |
| 201 | | Current Height | ______________ in cm |
|  | | Current Weight | ______________ in Kg |
|  | | Calculated BMI | _____________ (kg/m2) |
| 202 | | Cigarette smoking | 1. Never smoker  2. Former smoker  3. Current smoker |
| 203 | | Coffee | 1. Never  2. Daily  3. Occasionally |
| 204 | | Alcohol | 1. Never drinker  2. Former drinker  3. Current drinker |
| 205 | | Chat/khat | 1. Never chewer  2. Former chewer  3. Current chewer |
| 206 | | **Performance status: *Eastern Cooperative Oncology Group (ECOG) scale*** | |
|  | | 0. Fully active, able to carry on all pre-disease performance without restriction  1. Restricted in physically strenuous activity but ambulatory and able to carry out work of a light or sedentary nature, e.g., light house work, office work  2. Ambulatory and capable of all selfcare but unable to carry out any work activities. Up and about more than 50% of waking hours  3. Capable of only limited selfcare, confined to bed or chair more than 50% of waking hours  4. Completely disabled. Cannot carry on any selfcare. Totally confined to bed or chair | |
| 207 | | **Pain: *Brief pain inventory (BPI): Level of pain in the last 24 hours*** | |
| 207.1 | | Throughout our lives, most of us have had pain from time to time (such as minor headaches, sprains, and  toothaches). Have you had pain other than these everyday kinds of pain today? | 1. Yes 2. No |
| 207.2 | | Please rate your pain by marking the box beside the number that best describes your pain at its **worst**  in the last 24 hours. **[0: No pain;10: Pain as bad as you can imagine]** | 0 1 2 3 4 5 6 7 8 9 10 |
| 207.3 | | Please rate your pain by marking the box beside the number that best describes your pain at its  **least** in the last 24 hours. **[0: No pain;10: Pain as bad as you can imagine]** | 0 1 2 3 4 5 6 7 8 9 10 |
| 207.4 | | Please rate your pain by marking the box beside the number that best describes your pain on the **average**. **[0: No pain;10: Pain as bad as you can imagine]** | 0 1 2 3 4 5 6 7 8 9 10 |
| 207.5 | | Please rate your pain by marking the box beside the number that tells how much pain you have **right now**. **[0: No pain;10: Pain as bad as you can imagine]** | 0 1 2 3 4 5 6 7 8 9 10 |
| 207.6 | | What treatments or medications are you receiving for your pain? | ________________ |
|  | | Mark the box beside the number that describes how, during the past 24 hours, pain has interfered with your: [**0: does not interfere; 10: completely interferes**] | |
| 207.7 | | A. General Activity | 0 1 2 3 4 5 6 7 8 9 10 |
| 207.8 | | B. Mood | 0 1 2 3 4 5 6 7 8 9 10 |
| 207.9 | | C. Walking ability | 0 1 2 3 4 5 6 7 8 9 10 |
| 207.10 | | D. Norm al Work (includes both work outside the home and housework) | 0 1 2 3 4 5 6 7 8 9 10 |
| 207.11 | | E. Relations with other people | 0 1 2 3 4 5 6 7 8 9 10 |
| 207.12 | | F. Sleep | 0 1 2 3 4 5 6 7 8 9 10 |
| 207.13 | | G. Enjoyment of life | 0 1 2 3 4 5 6 7 8 9 10 |
| **PART III: CLINICAL AND DISEASE RELATED FACTORS (to be filled from registry record)** | | | |
| 301 | Types of admission | | 1. Outpatient  2. Inpatient |
| 302 | Cancer type | | 1. Breast  2. Cervical  3. Colorectal  4. Leukemia  5. Non-Hodgkin lymphoma  6. Other (please, specify)______________ |
| 303 | Time since diagnosis | | ___________ in months |
| 304 | Stage of cancer | | 1. Stage I  2. Stage II  3. Stage III  4. Stage IV  5. Stage not specified |
| 305 | Current treatment condition | | 1. Remission induction  2. Continuation  3. Maintenance |
| 306 | Current treatment modalities | | 1. Radiation therapy  2. Chemotherapy  3. Surgery  4. Chemo & radiation  5. Chemo & surgery  6. other:___________________ |
| 307 | Duration of treatment | | ______________ in months |
| 308 | Current medication use | |  |
| 308.1 | Analgesics | | 1. Yes 2. no |
| 308.2 | Antidepressants | | 1. Yes 2. no |
| 308.3 | Antiemetics | | 1. Yes 2. no |
| 308.4 | Anxiolytics | | 1. Yes 2. no |
| 308.5 | Corticosteroids | | 1. Yes 2. no |
| 308.6 | Diuretics | | 1. Yes 2. no |
| 308.7 | Other (please, specify) | | _________________ |
| 309 | Infections | | 1. Yes 2. no |
| 310 | Co-morbidity (hypertension/DM/Cardiac condition/anemia | | 1. Yes 2. no |
| **PART IV: THE BRIEF FATIGUE INVENTORY (BFI): level of fatigue in the past week** | | | |
| 401 | Throughout our lives, most of us have times when we feel very tired or fatigued. Have you felt unusually tired or fatigued in the past week? | | 1. Yes 2. no |
| 402 | Please rate your fatigue (weariness, tiredness) by circling the one number that best describes your fatigue **right now. [0: No Fatigue ; 10: Fatigue as bad as you can imagine]** | | 0 1 2 3 4 5 6 7 8 9 10 |
| 403 | Please rate your fatigue (weariness, tiredness) by circling the one number that best describes your **usual** level of fatigue during the past 24 hours. **[0: No Fatigue ; 10: Fatigue as bad as you can imagine]** | | 0 1 2 3 4 5 6 7 8 9 10 |
| 404 | Please rate your fatigue (weariness, tiredness) by circling the one number that best describes your **worst** level of fatigue during the past 24 hours. **[0: No Fatigue ; 10: Fatigue as bad as you can imagine]** | | 0 1 2 3 4 5 6 7 8 9 10 |
| 405 | Circle the one number that describes how, during **the past 24 hours**, fatigue has interfered with your [**0: does not interfere; 10: completely interferes**] | | |
| 405.1 | A. General activity | | 0 1 2 3 4 5 6 7 8 9 10 |
| 405.2 | B. Mood | | 0 1 2 3 4 5 6 7 8 9 10 |
| 405.3 | C. Walking ability | | 0 1 2 3 4 5 6 7 8 9 10 |
| 405.4 | D. Normal work (includes work outside the home and daily chores at home) | | 0 1 2 3 4 5 6 7 8 9 10 |
| 405.5 | E. Relations with other people | | 0 1 2 3 4 5 6 7 8 9 10 |
| 405.6 | F. Enjoyment of life | | 0 1 2 3 4 5 6 7 8 9 10 |

**END. Thank you!**
