# Supplementary material for: Prevalence of cancer related fatigue and its associated factors among adult cancer patients in eastern Ethiopia: A cross-sectional study
Source: PLoS One. 2026 Apr 24;21(4):e0347297. doi: 10.1371/journal.pone.0347297 (PMC13108774; doi:10.1371/journal.pone.0347297)
Supplement: Attachment — Submitted filename: Point- by-Point, CRF R1.docx [file pone.0347297.s003.docx]

**POINT -BY-POINT RESPONSE TO REVIEWERS**

**Title**: Prevalence of Cancer Related Fatigue and Its Associated Factors among Adult Cancer Patients in Hiwot Fana Comprehensive Specialized University Hospital, Eastern Ethiopia: A Cross-Sectional Study

**To:** PLOS ONE [Manuscript Number: PONE-D-25-44988]

| **Sections** | **Commitments** | **Responses** |
| --- | --- | --- |
|  | **Editor comments:** |  |
| General Comments: | The study addresses a significant, under-researched topic in a low-resource setting, a key strength. The manuscript is generally well-structured, the methodology is sound for a cross-sectional design, and the statistical analysis appears appropriate | Thank you. |
| Major Comments: | Statistical Output and Model Overfitting: The extremely high AOR for "Poor performance status" (30.01) with a very wide CI may indicate model overfitting, given the number of variables relative to the sample size. The authors should comment on model stability. Furthermore, the non-significant results and immense CIs for "Cancer type" suggest the model was underpowered for this variable. The authors should consider presenting a final model with only significant predictors or explicitly state this power limitation. | Thank you. The wider CIs were pertinent to “Performance status” and “cancer types”. This could be due to the smaller sample size in the group. However, we tried to investigate the model fitness (Hosmer-Lemeshow= 0.12) and analyze the multivariable assumptions, which were good fit. Nevertheless, rather than removing the variables we mentioned the prediction limitation under “limitation of the study”. (p 14) |
| Minor Comments: |  |  |
| 1. Abstract: | Results: The response rate in the abstract (90.5%) differs from the results section (96.4%). Please correct this inconsistency. | Thank you. Corrected. The right response is 90.5%.(p 7) |
| 2. Introduction/Background | The background is well-written. To strengthen it, consider adding a sentence or two specifically about the known risk factors for CRF in low-resource settings, if such literature exists, to better frame the study's contribution. | Thank you. We tried to include the local context. (p 3) |
| 3. Methods: | Study Participants and Sampling: The exclusion criterion "newly diagnosed patients" is noted. Please define "newly diagnosed" (e.g., within 1 month of diagnosis?) as this could significantly impact fatigue levels. | Thank you. In our study context newly diagnosed was considered as patients at first presentation or no prior follow up. Additional description was included. (p 4) |
| 4. Results | o Table 1: The category "Other" for religion has a percentage of "3", which seems to be a typo (likely 0.3%). o Flow of Participants: A participant flow diagram (CONSORT-style) would be helpful to clarify the numbers from the sampling frame (N=640) to selected (n=396? 422?) to the final analyzed (n=382). The text currently has some inconsistencies (e.g., 422 selected, but 396 selected on page 14). | -Thank you. Corrected as 0.3%. (p 8)  - Thank you. It was an error. The right sample size was 422 and the final result was analyzed by 382 (i.e response rate of 90.5%. Please look p 7.  - Thank you. A schematic presentation was included in the figures. |
|  | **Reviewer #1: Review Comments** |  |
| General Comments |  |  |
|  | -We apreciate the team work -Its obviious that cancer causes fatigu. Hence, why you are bothered to study it? Why you prefer Cevical cancer over other typ of cancer? Why you mixed from both the outptient and the inpatient?Who are those outpatient cases? | -Thank you. If we are not mistaken, this study focuses on cancer related fatigue among all types of cancer, not in particular to cervical cancer. We studied CRF to emphasis and indicate how prevalent it is, how impactful it could be in cancer care. In addition, we tried to identify what factors are significantly associated. |
|  | The abstacti is good but the backgroud i weak and the recomendations re nfakted. Again, it lacks key word. -Gramatical errors -Capitalzation isues .E.g Afan oromo -Mis used words E.g Addis Abeba -The service given by the hspital is misesd -Some of the sentence lack refrence | -Thank you. If we are not mistaken we tried to address the points made. Please look at p 2. |
| Specific Comments |  |  |
| 1.Abstract | Bakground is poor -The valdation of the tool is missed | -Thank you. We believe the validation of the tool was discussed at methodology section. Please, look at page 5. We can add in the abstract if suggested. |
| 2. Itroduction | -Fails to contain what it shoukd contain E.g. severity, factors asociated, eforts mae to prevent and contro it, hy cervical cancer i eced? | -Please, we need a bit clarification to reflect on |
| 3. Methods | -The study design and the samop8ng technique iis missed -Why you take 50% prevalnce to calcukte te samople size -Data quality assurance is not explicit E.g what do you mean brief orientation? -How did yu contro the social desirablity bias -What is the exact VIF value -Which was the exact outcome variable? | Thank you. In regard to study design and sampling technique, it was indicated at pages 4-5.  -The contents on brief orientation were indicted. Please look p 8.  - the VIF is < 1.4  -The outcome is CRF, little description is added. please look p 6 |
| 4 results | -Lacks some what logical flow -Add cross tabulations -Inconsiatent with the other section E.pian status, cancer stage | - Thank you. Please provide us specific suggestion. We will try to incorporate it.  - Cross tabulation was included in the factor analysis for significant variables. Please look pp 11-12. |
| 5. Discusion and othwr sections | -Weak -Be logical -Use upto date reference -Fas to use it o dat E.g female are high lkely to silufferfeom tfatigue can be xolnd by the fct that more womenn [n=239]. But yiu fai to exolan it. -Explaing and justify te main findings ith appropriate theoretical and practical implications -The reommendation ias bove th the of the study and general | - Thank you. Please provide us specific suggestion. We will try to incorporate it.  - We think we tried to provide scientific justification. Please look p 13. |
|  | **Reviewer #2:** |  |
|  | This is a well-conducted, hospital-based cross-sectional study assessing the prevalence and associated factors of cancer-related fatigue (CRF) among 382 adult cancer patients at Hiwot Fana Comprehensive Specialized University Hospital, Eastern Ethiopia. The study reports a high prevalence of clinically significant CRF (71.2%) and identifies several sociodemographic and clinical risk factors. The use of the validated Brief Fatigue Inventory (BFI) and systematic sampling strengthens the methodology. The findings are novel for the region and have clear clinical and public health implications. With weakness for revisions, to be considered suitable for publication: | -Thank you. |
| 1. Abstract & Results Inconsistency | • Abstract states: "private occupational status (AOR=6.44, 95%CI: 2.42-17.12)" • But in Page 1: "private occupational status (AOR=6.44, 95%CI: 2.42-17.12)" is correct, while Page 8 (line 44) says "never used coffee (AOR=7.02, 95%CI: 2.37-20.75)" — consistent. • However, in Page 1 (Results): "unclassified cancer stages (AOR=4.84, 95%CI: 1.42-16.57)" vs. Page 8 (line 47): "unclassified cancer stages (AOR=4.84, 95%CI: 1.42-16.57)" — consistent. → No inconsistency, but standardize formatting across abstract, results, and discussion. Recommendation: Use one decimal place for all AORs and CIs throughout (e.g., AOR=6.4, 95%CI: 2.4–17.1). Currently mixed (6.44 vs 7.02 vs 4.68). | -Thank you. The argument to use one decimal number could enhance readability and prevents confusion. However, to the best of our knowledge using two decimal points were conventionally accepted in medical journals and balances readability and precision. Thank you |
| 2. Coffee Consumption – Protective Association | • Finding: "never used coffee (AOR=7.02, 95%CI: 2.37–20.75)" → strong protective effect implied. • This is biologically plausible (caffeine as CNS stimulant), but not discussed in limitations or prior literature. Recommendation: • Add 1–2 sentences in Discussion citing possible mechanisms (e.g., caffeine’s effect on adenosine receptors, alertness, mood). • Reference studies (if any) on caffeine and fatigue in chronic illness. • Retain call for further research — appropriate and justified. | -Thank you. The recommendations and the justifications provided are plausible. As we tried to respond and stated in the justification, the caffeine in the coffee could be the possible reason. Please look p 14. However, to the best of our literature review ability and access, only study on mice was reported. |
| 3. Cancer Stage Classification | • "Unclassified" stage has AOR=4.84 (1.42–16.57) — significant. • But no explanation of what "unclassified" means (e.g., missing records? late presentation? diagnostic limitations?). Recommendation: • Add one sentence in Methods or Results: "Unclassified stage refers to patients without documented staging due to incomplete diagnostic workup or late presentation." | -Thank you. It is corrected. Please look p 6. |
| 4. Language, Grammar & Typos (Minor) need to be revised and corrected |  | -Thank you. We tried to explore and corrected it. |
| 5. Tables/Figures (Not Provided) | • Manuscript refers to Supporting Information files for data. • But no tables in the draft (e.g., sociodemographic characteristics, bivariate results). Recommendation: • Include at least one table in main manuscript or SI: o Table 1: Participant characteristics (n, %) o Table 2: Bivariate and multivariable logistic regression results | -Thank you. We think we did include and provided table in the main document and figures in separated document. Please look pp 8-9, 11. |
| 6. Limitations Section Missing- No explicit Limitations subsection. | Recommendation: Add under Discussion: "This study is limited by its cross-sectional design, which precludes causality. Self-reported data may introduce recall bias. The single-center setting may limit generalizability. Coffee consumption was assessed crudely (ever/never); frequency and amount were not evaluated." | - Thank you. Limitation section was paraphrased as recommended. Please look p 14. |
|  | **Reviewer #3:** |  |
|  | The manuscript is Highly Suitable for publication in PLOS ONE.The study addresses Cancer Related Fatigue (CRF), a highly prevalent and distressing symptom in oncology 1111, but examines it in a context where evidence is scarce (Eastern Ethiopia). The finding of a high prevalence rate (71.2%) is clinically significant 3, and the identification of unique local associated factors (e.g., never used coffee, private occupational status, rural residence) makes a genuine contribution to supportive oncology research4. | - Thank you. |
| Acronym List Error: | The abbreviation list contains a typo: "CFR" is listed for "Cancer Related Fatigue" 5, but the correct acronym used throughout the paper is CRF. The authors should ensure the list uses CRF. | -Thank you. Corrected. Please look p 15 |
| Statistical Reporting in Abstract: | The Abstract reports Adjusted Odds Ratios (AOR) but includes the initial of the factor in the CI: AOR=6.44, 95%CI 1:2.42-17.12 for private occupational status77. This initial (1:) is likely a formatting artifact (possibly from transcription) or a reference label and should be removed or corrected to standard format: 95% CI (2.42-17.12).Methodology Clarity (Sample Size): In the methods section, the sample size calculation initially uses P as 50% but then shows an intermediate step using P=0.748. This 0.748 should be clearly defined or referenced in the calculation steps (it likely represents the prevalence used from a prior study, which is mentioned as a comparable study with 74.8% prevalence 9). The final sample size determination based on the 74.8% prevalence should be explicitly stated for clarity. n=(1.96)^2 * 0.748(1-0.748) / (0.05)^2 = 284 (There may be an issue with calculation or transcription as the document reports 38410, likely stemming from a previous P=50%. The authors should verify and state the source of the prevalence used for n=384). Correction/clarification is vital here. | -Thank you. That might be due to window incompatibility. The formatting is fine in our document.  -Thank you. The correct p used was 50% and corrected. Please, look p 5. |
| Discussion Flow: | In the Discussion when linking to external research, the phrase "This is in line by extension with a study" 11 (referring to coffee use) could be rephrased for better academic flow (e.g., "This finding is indirectly supported by a study..." or similar). | -Thank you. Corrected.  Please look p 14 |
