# Supplementary material for: Prevalence of cancer related fatigue and its associated factors among adult cancer patients in eastern Ethiopia: A cross-sectional study
Source: PLoS One. 2026 Apr 24;21(4):e0347297. doi: 10.1371/journal.pone.0347297 (PMC13108774; doi:10.1371/journal.pone.0347297)
Supplement: Attachment — Submitted filename: Point- by-Point, CRF R2.docx [file pone.0347297.s004.docx]

**POINT -BY-POINT RESPONSE TO REVIEWERS**

**Title**: Prevalence of Cancer Related Fatigue and Its Associated Factors among Adult Cancer Patients in Hiwot Fana Comprehensive Specialized University Hospital, Eastern Ethiopia: A Cross-Sectional Study

**To:** PLOS ONE [Manuscript Number: PONE-D-25-44988]

| **Sections** | **Commitments** | **Responses** |
| --- | --- | --- |
|  | Additional Editor Comments: |  |
|  | Dear author,   address the comments and questions raised by the reviewer. | Thank you, responses were provided. |
|  | Reviewer #1: Review Reports |  |
|  | A. General Comments ♣ The manuscript seems the outcome of collective efforts and we appreciate team spirit in health research. In future studies think about gender representation. ♣ Grammar and language E.g. In the key words in ‘eastern”, ‘e’ is written in lower case. Use ‘Eastern” throughout the document. Additionally, Afan Oromo is written as ‘Afan-oromo’ ♣ Incomplete sentence ♣ Use of future tense ♣ The way you write numbers when in thousands ♣ Inconsistent E.g. compare the abstract with the entire document. | -Thank you. We will consider it for future studies.  - Thank you. We tried to correct the grammatical and language concerns. For further edition, please provide us specific needs for correction. |
|  | B. Specific Comments |  |
| i. Scope, Title and authors: | It is too long and try to shorten it. • If systematic review was done in Ethiopia [ see line 297], why did you do this study? • The number of authors and their email is mismatched. | -Thank you. Little modification was made in the title.  - Thank you. However, the systematic review was conducted targeting developed countries and Chinese populations, not Ethiopian and the focus was only colorectal cancer patients.  - Thank you. We hope the number of authors were in line with the email addresses. The email were sequenced horizontally. |
| ii. Abstract | Cancer should be rewritten as “various types of cancer patients” in line 41. In the background section the term “nevertheless” is not an important word and flawed the meaning of your manuscript. Therefore, it is better if you could change it. The methods fail how the data is presented. In the result section you stated “clinical significant cancer related fatigue”. Is this type of fatigue, the type of fatigue you are dealing in the background section? What does “integrated supportive care service” means in line 49 and is that in line with the objective of the study? | -thank you. Corrected  - Thank you. Corrected  -thank you. Clinical significant fatigue indicates the level or severity of fatigue worthy for clinical intervention. However, to prevent confusion among readers, the terms is removed from the abstract section.  - Thank you. The statement is modified with better expressions. |
| iii. Background | o Fails to define fatigue and cancer related fatigue. If I were you, I will write as: definition, types, causes, magnitude, severity, factors, consequences, initiatives to prevent and control fatigue be it pharmacological or non-pharmacological, then the gaps then the objectives? o Not described the global and regional prevalence of fatigue and how fatigue is created by cancer [pathophysiology]. o Some lacks reference E.g. line 63 ‘Unlike fatigue among healthy individuals, CRF is more severe and is persistent in spite of rest.’ o In line 58, if “fatigue, tiredness and exhaustion” were interchangeable names, who permitted you to use fatigue? And who do you think is the best institution to nomenclature it? o Over referencing E.g. reference 4. o What does “Globally CRF is highly prevalent” means in line 70? o Logical flow is not intact. o “However, there is a paucity of evidence” in line 86 is exaggerated> hence, try to use other synonyms. o The paragraphs are not well explained and revisit it. | -thank you. We tried to revise and incorporate the comments accordingly.  -Thank you. We tried to include the pathophysiology briefly.  -Thank you. We tried to revise the sequence and flow of the paragraphs accordingly. Please, look the tracked version. |
| iv. On the Methods | ¬ How do you differentiate fatigue that is entirely resulted from cancer and other diseases? ¬ Is that fatigue among patients on treatment or not? Is that new or old? Is that benigh or malignant? Who diagnosed? Who finally declared and what is his profession? Is that entirely clinical or have laboratory investigation? ¬ The inclusion criteria: E.g. How long was needed to define cancer and fatigue? ¬ The study setting should also explain the type of services rendered for the community particularly related to cancer? ¬ Source and study participants were missed. ¬ The inclusion criteria are not presented. ¬ If there are studies in Hawassa and Amhara region [you should state the particular area], why you used 50% to calculate sample size? ¬ Who introduced Brief Fatigue Inventory (BFI ) tool to Ethiopia? Where? Is that validated? If validated is that for Amharic or Afan Oromo language? What about the reliability and validity of the tool. Similarly, respond also to Oslo social support scale? ¬ What do you think is the main limitation of systematic sampling and how did you overcome it? ¬ Surprisingly enough, the data quality assurance method is not written. ¬ The outcome variable should be “cancer related fatigue” line 151. ¬ How many outcome variables did you have? ¬ What does unclassified means if you are collecting the data from the oncology unit? ¬ Sum score is missed in the data processing and analysis sub section. ¬ Why did you use both median and mean? Line 171. ¬ What was done for those who have severe fatigue or in case ‘uncontrolled fatigue’? ¬ How was social desirability bias? ¬ How do you compare fatigue of the one who is admitted due to the progression of the cancer with ambulatory outpatient patients? | -To the best of our knowledge, comorbidities including anemia and infections are common among cancer patients due to the cancer or the treatment side effects, In which difficult to rule out. To minimize the confounding we tried to include since cancer confirmation and we did multivariable analysis considering them as independent factors.  -all cancer patients with confirmed pathological report and at least with one follow up were considered in the study.  -thank you. We tried to incorporate it under study participant and sampling techniques sections. Thank you  -the BFI tool was validated in the Amharic version. Please, look the reference. The psychometric properties were described under the data collection tool section.  -thank you. The data quality assurance measures is included in the revision.  -thank you. We tried to revise it accordingly.  -thank you. To our understanding the difference in fatigue level between impatiens and outpatients may be explained in clinical status and provided clue for external validity of the tool. |
| Results | o Relatively it is good. But you have presented few variables? o What is you base to classify the income? o Check for the sum o The result revealed no new findings because you should check the nature of the included participants E.g./\. Most of the respondents were female, dwell in urban area, most were married, o Cross tabulation is my recommendation. o Highlight or bold the main findings in the table E.g. Table 3. o The wy you report the odds ratio needs revisit E.g. In the result and discussion section. | -thank you.  -thank you. We tried to check the normal distribution of the incomes and then since it is normally distributed we used the men value.  -Thank you. P-values re bolded |
| Vi. Discussion and the consequent sections | ♣ Again, state your findings, then compare with the contemporary studies, justify appropriately, explain in detail and refer up-to-date references. For instance, explain “This might be due to the socio-demographic variation, outcome measurement tools.” In line 262. In the third paragraph, when justifying rural residence it is surprising not to mention travel distance and transportation. ♣ Revisit paragraph 2 and it begins in a wrong way. ♣ What does “had private occupation” means in line 279. In this sentence check the language and which private occupation specifically? ♣ In line 281 you stated “This might be due to job insecurity”. How do you reconcile with the effort of the government to embrace them? ♣ Is this new “As for the stage of cancer, those participants with advanced stages (III & IV) and unclassified stages were more likely to report cancer related fatigue as compared to participants with early stages (I & II).”? in line 295-297. ♣ Can a cancer patient will fail to recall pain since it is more of feeling. [see line 302] ♣ You have already told that your study is statistically insufficient line 303. Therefore, why do you send it for publication and how did you overcome it? ♣ Line 312-313 “High risk groups such as female, rural residents, inpatients, private workers might require tailored interventions. is wrong”. Because this had occurred because they are over sampled form the beginning of the study. ♣ In the acknowledgement what does “their relentless support.’ Means? [ see line 337] ♣ I cannot hide my humor a paper dealing regarding cancer in Ethiopia fails to cite the private, government [ for instance MOH-Ethiopia and associations role or initiatives regarding the expansion of the service, development of guidelines, expansion of oncology centers in the country. Regards, | -Thank you. We tried to follow the logical flow of the paragraphs.  -Thank you. It is included.  -thank you. Private occupation indicted employed participants in private industries/companies regardless.  -thank you. We don’t think it introduced new findings  -thank you. We tried to mention the cross-tabulation finding for poor performance status that resulted the wider CI, not the total sample size.  -thank you. We don’t think the result is influenced by the sample size. Some are almost proportional in size and to prevent the influence of sample size randomness and multivariable analysis were assured and performed.  -thank you. It would be paramount to include the national initiatives and guidelines, however, we fail to mention the system level analysis due to our focus and scope in symptom management and patient level outcome |
